# Exposure to bisphenol A enhanced lung eosinophilia in adult male mice

**DOI:** 10.1186/s13223-016-0122-4

**Published:** 2016-04-14

**Authors:** Miao He, Takamichi Ichinose, Seiichi Yoshida, Hirohisa Takano, Masataka Nishikawa, Takayuki Shibamoto, Guifan Sun

**Affiliations:** Environment and Non-communicable Disease Research Center, School of Public Health, China Medical University, Shenyang, 110122 China; Department of Health Sciences, Oita University of Nursing and Health Sciences, Oita, 870-1201 Japan; Environmental Health Division, Department of Environmental Engineering, Graduate School of Engineering, Kyoto University, Kyoto, 615-8530 Japan; Environmental Chemistry Division, National Institute for Environmental Studies, Ibaraki, 305-8506 Japan; Department of Environmental Toxicology, University of California, Davis, CA 95616 USA

**Keywords:** Bisphenol A, Lung eosinophilia, Mice, Macrophage

## Abstract

**Background:**

Bisphenol A (BPA) is useful in many manufacturing processes and is also found in commonly used consumer products. Previous experimental studies have reported that perinatal exposure to BPA promotes the development of allergic lung inflammation in childhood and even into adulthood. In this study, the effects of BPA on allergic lung inflammation in adults were investigated in murine lungs.

**Methods:**

CD-1 mice were orally administrated with 1 mg of BPA/mouse four times at one-week intervals with or without ovalbumin (OVA). The pathologic changes in the airways, cytological alterations in bronchoalveolar lavage fluid (BALF), levels of inflammatory cytokines/chemokines in BALF, and OVA-specific IgE and IgG1 antibodies in serum were measured in the treated CD-1 mice. In vitro study using RAW264.7 cells, which are macrophage-like cells derived from BALB/c male mice, was conducted. The gene expression of cytokines and chemokines were measured.

**Results:**

BPA enhanced eosinophil recruitment induced by OVA in the alveoli and in the submucosa of the airway, which has a goblet cell proliferation in the bronchial epithelium. BPA increased Th2 cytokines-interleukin-13 (IL-13), eosinophil-relevant cytokines and chemokines, such as IL-5, and CCL2 induced by OVA, in BALF. BPA induced adjuvant effects on OVA-specific IgG1 production. In the in vitro study using RAW264.7 cells, BPA increased the mRNA expression of IL-1β, IL-6, CCL2 and CCL3 compared with the control and OVA groups.

**Conclusions:**

These results suggest that (1) the exposure of BPA could synergize with an OVA challenge to aggravate the severity of lung eosinophilia in adult mice, possibly by promoting a Th2-biased immune response and (2) the activation of macrophages and inflammatory cytokines released from these cells by BPA could be participating in this phenomenon.

## Background

Rapid industrialization and improvement in living standards come along with an increase in toxic hazards. Bisphenol A (4,4′-(propane-2,2-diyl) diphenol or BPA), stable in sediment and detectable in almost all bodies of water and food, is an intermediate in the production of polycarbonate and epoxy resins [1]. Thus, wildlife and human exposure to BPA is inevitable, and is likely to continue and even to increase.

Allergic asthma is an airway inflammatory disease that is characterized by bronchial hyper-responsiveness, airway eosinophilia, goblet cell hyperplasia and production of allergen specific immunoglobulin (Ig) [2]. The prevalence of asthma in developed countries is approximately 10 % in adults and even higher in children, whereas in developing countries, the prevalence is lower but increasing rapidly [3]. Previous studies have reported that maternal or fetal exposure to BPA exacerbated allergic sensitization and bronchial inflammation and responsiveness in a susceptible-animal model of asthma [4, 5]. Asthma patients are exposed to BPA everyday via food, water and air. However, to our knowledge, few experimental studies have documented the aggravating effects of BPA on allergic asthma in adults.

In the present study, the effects of the BPA used in the inner lining of metal cans on ovalbumin (OVA)-induced lung eosinophilia were examined in adult mice. Pathologic changes in airway, cytological alterations in bronchoalveolar lavagefluids (BALF), changes of inflammatory cytokines and chemokinesin BALF, and enhancement of IgE and IgG1 in serum were investigated.

Macrophages are innate immune cells that play a critical role in the early phases of host defense against pathogens, coordination of the adaptive immune response, and regulation of inflammation and tissue repair. Through activation signals by environmental cues and various ligands, macrophages may change their polarization state, leading to altered immune responses [6]. Macrophages produce a variety of cytokines and mediators that are vital for immune and inflammatory responses in their response in response to external agents [7]. A previous study has presented evidence supporting the claim that BPA plays a role in the modulation of macrophage function through their ability to enhance tumor necrosis factor (TNF) and interleukin (IL)-6 expression [8].

In the in vitro study, the gene expression of cytokines and chemokines in RAW264.7 cells, which are macrophage-like cells derived from BALB/c male mice, was measured in the presence of BPA and/or OVA.

## Methods

### Animals

Male CD-1 mice (5 weeks of age) were purchased from Charles River Japan, Inc. (Kanagawa, Japan). Mice were checked for abnormal body weight or sickness for 1 week, and then 48 mice were used at 6 weeks of age. They were fed a commercial diet CE-2 (CLEA Japan, Inc., Tokyo) and given water ad libitum./Mice were housed in plastic cages lined with soft wood chips. The cages were placed in an air conditioned room at 23 °C with 55–70 %/humidity and a light/dark (12 h/12 h) cycle. CD-1 male mice were used because of their moderate responsiveness to allergic airway inflammation caused by OVA [9]. The study adhered to the US. National Institutes of Health Guidelines for the use of experimental animals. The animal care method was approved by the Animal Care and Use Committee at Oita University of Nursing and Health Sciences in Oita, Japan.

### Study protocol

**Fig. 1:**
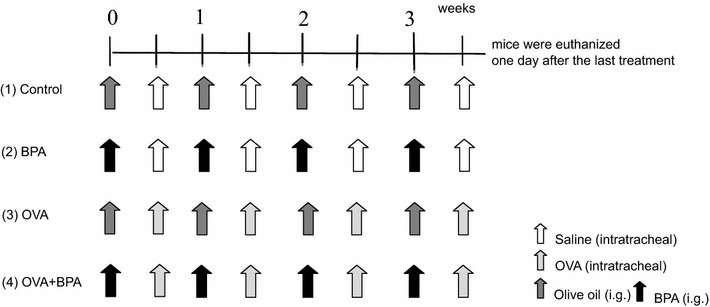
Experimental protocol. *1* Control: orally administrated with 0.2 mL olive oil and instilled intratracheally with 0.1 mL of normal saline per mouse four times at one-week intervals; *2* BPA: orally administrated with 1 mg/0.2 mL BPA and instilled intratracheally with 0.1 mL of normal saline per mouse four times at one-week intervals; *3* OVA: orally administrated with 0.2 mL olive oil and instilled intratracheally with 1 μg/0.1 mL OVA per mouse four times at one-week intervals; *4* OVA + BPA: orally administrated with 1 mg/0.2 mL BPA and instilled intratracheally with 1 μg/0.1 mL OVA per mouse four times at one-week intervals

A total of 48 male CD-1 mice were divided into four groups (n = 12 per group) according to treatment protocol: control, BPA, OVA and OVA + BPA. BPA (Sigma-Aldrich, St Louis, MO) was dissolved in ethanol (Otsuka Co, Kyoto, Japan), and then diluted in olive oil (final ethanol concentration 5 %) for oral administration. The instillation dose of BPA was 1 mg per mouse. The dose of BPA used in this study was designed to be an order of magnitude lower than the established maximum nonlethal threshold in rodents (200 mg/kg BW/d) [10]. Mice were orally administrated with 1 mg BPA/0.2 ml olive oil or 0.2 ml olive oil (final ethanol concentration 5 %) four times at one-week intervals (Fig. 1).

OVA (A7641: Grade VII) was purchased from Sigma-Aldrich (St. Louis, MO). OVA was dissolved in sterile saline (0.9 % NaCl, LPS free) for injection (Otsuka Co, Kyoto, Japan); in accordance with previous reports [9, 11, 12], the instillation dose was 1 μg per mouse. Four instillations, with or without OVA, were administered at one-week intervals (Fig. 1). Mice were anesthetized with 4 % halothane (Takeda Chemical, Osaka, Japan) and intratracheally instilled with OVA or sterile saline (Otsuka Co., Kyoto, Japan) through a polyethylene tube under anesthesia with 4 % halothane (Takeda Chemical, Osaka, Japan). One day after the last administration, mice from all groups (age = 9.5 weeks) were euthanized by exsanguination under deep anesthesia by intraperitoneal injection of pentobarbital (Fig. 1). Total duration of the experiment is 3 weeks and a half.

### Pathological evaluation

Six of the 12 mice from each group were used for pathologic examination. Lungs were fixed in 10 % neutral phosphate-buffered formalin. After separation of the lobes, 2 mm thick blocks were taken for paraffin embedding. Embedded blocks were sectioned at a thickness of 3 μm, and were stained with hematoxylin and eosin (H and E) to evaluate the degree of infiltration of eosinophils or lymphocytes in the airway from proximal to distal. Sections were also stained with periodic acid-Schiff (PAS) to evaluate the degree of proliferation of goblet cells in the bronchial epithelium. Pathological analysis of the inflammatory cells and epithelial cells in the airway of each lung lobe on the slides was performed using a Nikon ECLIPSE light microscope (Nikon Co, Tokyo, Japan).

### Bronchoalveolar lavage fluid (BALF)

The remaining six mice were used to examine the free cell contents from BALF. BALF and cell counts were conducted by a previously reported method [9, 11, 12]. In brief, tracheas were cannulated after the collection of blood. The lungs were lavaged with two injections of 0.8 ml of sterile saline at 37 °C by a syringe. The lavaged fluid was harvested by gentle aspiration. The mean volume retrieved was 90 % of the amount instilled (1.6 ml). Fluids from the two lavages were pooled, cooled to 4 °C, and centrifuged at 1500 rpm for 10 min. The total amount of lavages collected from individual mice was used to measure the protein levels of cytokines and chemokines in the BALF. The total cell count of fresh fluid specimen was determined using a hemocytometer. Differential cell counts were assessed on cytologic preparations. Slides were prepared by Cytospin (Sakura Co, Ltd., Tokyo, Japan) and stained with Diff-Quik (International Reagents Co, Kobe, Japan). A total of 300 cells were counted under oil immersion microscopy. The BALF supernatants were stored at −80 °C until analyzed for cytokines.

### Quantitation of cytokines in BALF

The cytokine protein levels in the BALF were determined using enzyme-linked immunosorbent assays (ELISA). IL-1β, IL-6, IL-13, keratinocyte chemoattractant (KC or CXCL1), monocyte chemotactic protein-1 (MCP-1 or CCL2) and macrophage inflammatory protein-1α (MIP-1α or CCL3) were measured using ELISA kits from R and D Systems Inc. (Minneapolis, MN). IL-5 was measured using ELISA kits from Endogen (Cambridge, MA).

### Antigen-specific IgE and IgG1 antibodies

OVA-specific IgE and IgG1 antibodies were measured using a Mouse OVA-IgE ELISA kit and a Mouse OVA-IgG1 ELISA kit (Shibayagi Co, Shibukawa, Japan). According to the manufacturer’s protocol, 1 U of anti-OVA IgE is defined as 1.3 ng of antibody, and 1 U of anti-OVA IgG1 as 160 ng of antibody. The absorption at 450 nm (sub-wave length, 620 nm) for OVA-specific IgE and IgG1 antibodies was measured using a microplate reader (Spectrafluor, Tecan, Salzburg, Austria).

### Cell culture

RAW264.7 cells, which are macrophage-like cells derived from BALB/c male mice, were cultured at 37 °C in a humidified atmosphere of 5 % CO_2_–95 % air and maintained in Dulbecco’s modified Eagle’s medium with 10 % heat inactivated fetal bovine serum. For gene expression analysis, the cells were plated at a concentration of 4 × 10^5^ cells per 60-mm dish, and then PBS, OVA and OVA + BPA were added to cells to give BPA final concentrations of 0.5 and 5 μg/ml, respectively. Cells were then incubated for 12 h.

### Gene expression analysis

Total RNA was extracted by standard procedures using 0.5 ml of Isogen (Nippon Gene) per dish. After DNase treatment of the total RNA, cDNA was synthesized by reverse transcription using Moloney murine leukemia virus (M-MLV). Quantitative polymerase chain reactions (PCR) were performed using an ABI Prism 7000 Sequence Detection System (ABI) under the same conditions as in previous studies [11, 13]. Two wells were used for each sample. The relative expression of each sample was calculated as the mean value divided by the mean value for GAPDH. The primers and probes used in this in vitro study are shown in Table 1.

**Table 1:** Primers and probes used in this in vitro study

Primers and probes	Gene sequence
GAPDH sense	TGCACCACCAACTGCTTAG
GAPDH antisense	GGATGCAGGGATGATGTTC
GAPDH probe	CAGAAGACTGTGGATGGCCCCTC
IL-1β sense	TCCTGAACTCAACTGTGAAATGC
IL-1β antisense	AGCCCAGGTCAAAGGTTTGG
IL-1β probe	AGCAGCCCTTCATCTTTTGGGGTCCG
IL-6 sense	CCGGAGAGGAGACTTCACAGA
IL-6 antisense	GTTGTTCATACAATCAGAATTGCCATT
IL-6 probe	ACCACTCCCAACAGACCTGTCTATACCACT
CCL2 sense	TCTGGGCCTGCTGTTCACA
CCL2 antisense	CCAGCCTACTCATTGGGATCA
CCL2 probe	TTGGCTCAGCCAGATGCAGTTAACGC
CCL3 sense	ATTCCACGCCAATTCATCGT
CCL3 antisense	TTGGAGTCAGCGCAGATCTG
CCL3 probe	CCTTTGCTCCCAGCCAGGTGTCATT

### Statistical analysis

Statistical analyses of cell numbers and cytokine proteins in BALF, IgE and IgG1 in serum and of gene expression in RAW264.7 cells were conducted using the Tukey Test for Pairwise Comparisons (KyPlot Ver.5, Kyens Lab Inc., Tokyo, Japan). The Tukey test is essentially a *t* test, except that it corrects for experimental error rate (when there are multiple comparisons being made, the probability of making a type I error increases—the Tukey test corrects for that, and is thus more suitable for multiple comparisons than doing a number of *t* tests would be). Differences among groups were statistically significant when p < 0.05.

## Results

### BPA increased BALF cell numbers

**Fig. 2:**
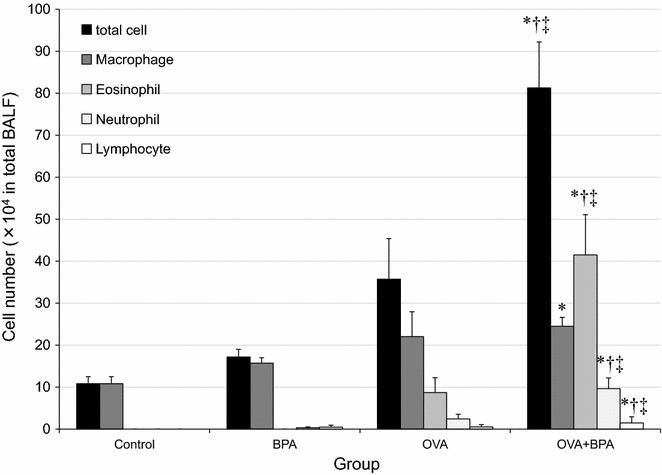
Cellular profile in bronchoalveolar lavage fluids (BALF). All values were expressed as mean ± SEM. *p < 0.05 vs. Control, ^†^p < 0.05 vs. BPA, ^‡^p < 0.05 vs. OVA

To evaluate the effect of BPA on lung inflammation induced by OVA, the cellular profile of BALF 24 h after the last intratracheal instillation was investigated (Fig. 2). BPA and OVA alone caused no significant increase in the number of macrophages, neutrophils, eosinophils or lymphocytes compared with the controls. However, the administration of OVA combined with BPA resulted in a remarkable increase in the number of macrophages, neutrophils, eosinophils and lymphocytes compared with controls, BPA and OVA alone (p < 0.05).

### BPA enhanced pathologic changes in the airway

**Fig. 3:**
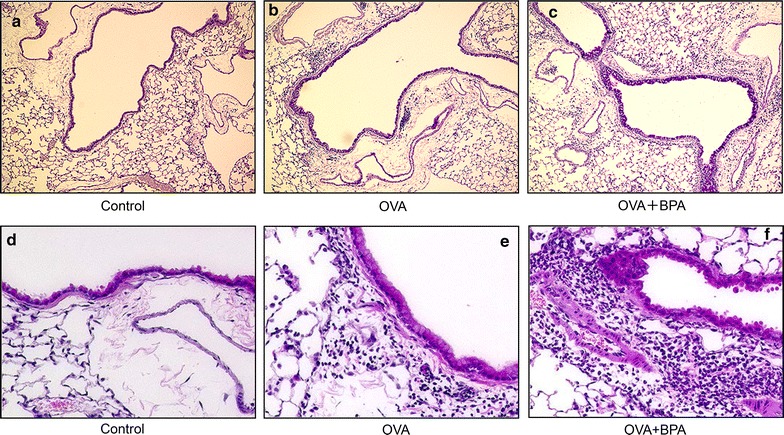
Effects of BPA on pathological changes in the lungs. **a** and **d** No pathological changes in lungs treated with saline. **b** Slight proliferation of goblet cells that have mucus stained pink with PAS solution in the airway epithelium treated with OVA alone. **c** Marked proliferation of goblet cells and numerous inflammatory cells in the airway treated with OVA + BPA. **e** Slight infiltration of eosinophils and lymphocytes into the airway submucosa treated with OVA alone. **f** Marked infiltration of eosinophils and lymphocytes into connective tissue in the airway treated with OVA + BPA. **a**–**c** PAS stain; **c**–**e** HE stain; ×200

To confirm the effects of BPA on airway inflammation and goblet cell proliferation caused by OVA, the lung pathology of the mice was examined (Fig. 3). No pathologic alterations were found in the lungs of the control group (Fig. 3a, d). Treatment with OVA alone caused slight goblet cell proliferation (Fig. 3b) in the bronchial epithelium. The combined instillation of OVA and BPA significantly increased goblet cell proliferation in the airway and caused prominent infiltration of eosinophils and lymphocytes to appear in the connective tissues of the airways (Fig. 3c, f).

### BPA enhanced cytokine and chemokine levels in BALF

**Fig. 4:**
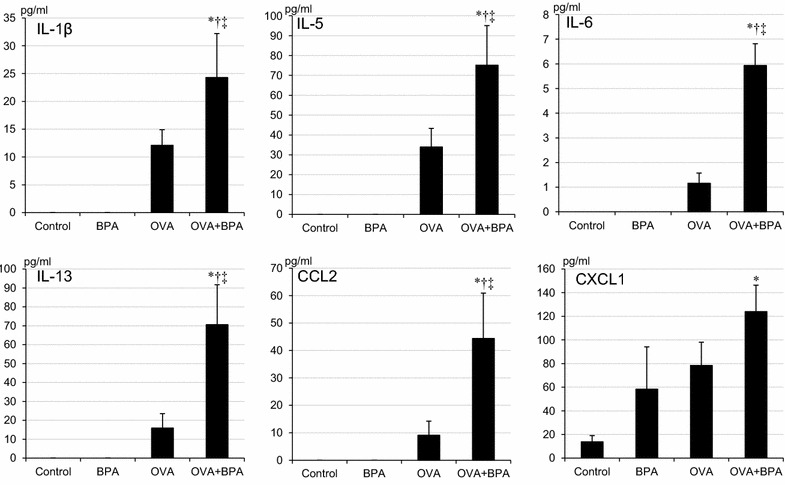
Cytokines and chemokines expression in bronchoalveolar lavage fluids (BALF). All values were expressed as mean ± SEM. *p < 0.05 vs. Control, ^†^p < 0.05 vs. BPA, ^‡^p < 0.05 vs. OVA

To investigate the effects of BPA on OVA-induced lung eosinophilia, the protein levels of cytokines and chemokines in BALF were measured (Fig. 4). OVA + BPA increased CXCL1 compared with the control group (p < 0.05). OVA + BPA significantly increased IL-1β, IL-6 and CCL2 compared with the controls, the BPA and the OVA groups (p < 0.05). Furthermore, OVA + BPA significantly increased eosinophil-produced IL-5 and Th-2 relevant cytokine IL-13 compared with the other groups (p < 0.05).

### BPA enhanced serum OVA-specific IgE and IgG1

**Fig. 5:**
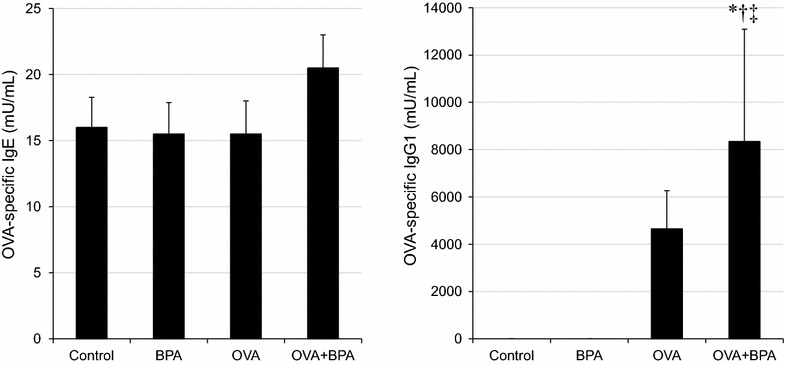
OVA-specific IgE and OVA-specific IgG1 production in serum. All values were expressed as mean ± SEM. *p < 0.05 vs. Control, ^†^p < 0.05 vs. BPA, ^‡^p < 0.05 vs. OVA

To examine whether BPA had adjuvant activity on antigen specific Ig production, OVA-specific IgE and IgG1 antibodies were measured (Fig. 5). Co-treatment with OVA and BPA significantly increased OVA-specific IgE and IgG1 production compared with OVA group (p < 0.05).

### Cytokine secretion from RAW264.7 cells by BPA

**Fig. 6:**
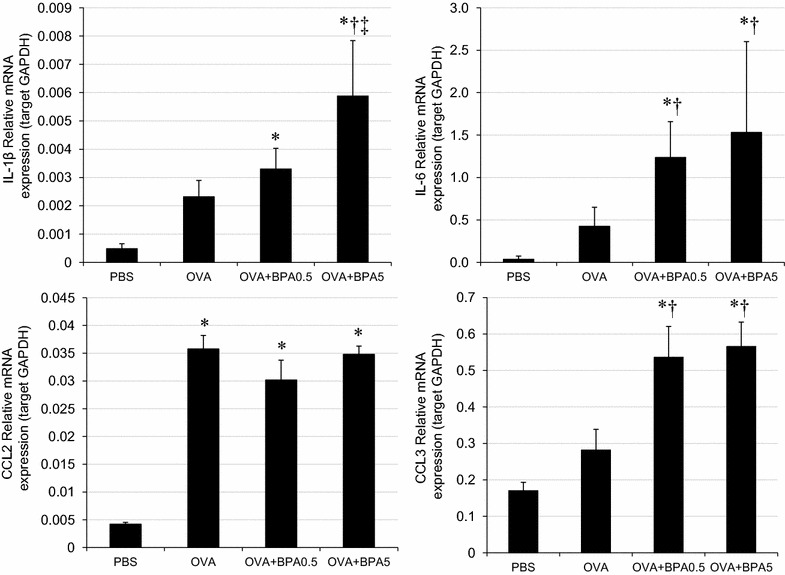
mRNA expression in RAW264.7 mouse macrophage cells. All values were expressed as mean ± SEM. *p < 0.05 vs. Control, ^†^p < 0.05 vs. OVA, ^‡^p < 0.05 vs. OVA + BPA0.5

To evaluate secretion of pro-inflammatory cytokines from RAW264.7 cells associated with BPA treatment, the mRNA expression levels of IL-1β, IL-6, CCL2 and CCL3 were measured (Fig. 6). BPA significantly increased the expressions of IL-1β, IL-6 and CCL3 compared with PBS and OVA treatments (p < 0.05). Furthermore, expression of IL-1β in OVA + BPA_5_ was higher than that of OVA + BPA_0.5_ (p < 0.05).

## Discussion

Although in recent years several experimental studies have addressed the question, of whether BPA may affect the development and the outcome of allergic diseases, the overall picture has still not become clearer. Some studies demonstrated a BPA-induced increase of Th2-driven immune reactions with increased IgE and IL-4 cytokine levels, and showed an augmentation of Th1-mediated responses with rather decreased IgE levels [14]. In other studies, maternal BPA exposure came into focus, since it has been suggested that the prenatal period represents a critical window for the developing immune system to be primed towards a specific immune phenotype [15]. However, the data concerning prenatal BPA exposure are diverse, ranging from increased asthma susceptibility [4] to an elevated Th1 immune response [14] or to no effects [16]. These striking differences might be caused primarily by the BPA doses used in the various studies, which ranges from 0.5 to 5000 mg/kg/day, or by diverse exposure routes, resulting in different bioavailability and metabolism of BPA [17]. In the present study, the immunotoxicity of BPA in adult male mice were investigated.

The results of this study suggest that co-exposure to BPA and OVA caused increases in neutrophil and eosinophil accumulation in BALF, and eosinophil infiltration and proliferation of goblet cells in the airway, which are effects pathologically similar to human asthma. There were also remarkable increases in the expression of proinflammatory cytokines (IL-1β, IL-5, IL-6 and IL-13), and chemokines (KC and MPC-1) in BALF, suggesting that co-exposure may cause increased neutrophil and eosinophil accumulation via the remarkable increases of these pro-inflammatory mediators.

The Th2 cytokine IL-5 stimulates the differentiation of bone marrow cells to form eosinophils, which are then activated and induced by eotaxins to migrate into inflammatory tissue [18]. IL-13, also released from Th2 lymphocytes, stimulates B cells, induces the production of antigen specific antibodies [19, 20], and promotes mucous secretion and the production of mucous cells, such as goblet cells, in the bronchial epithelium [21]. Our study indicates that the co-treatment of OVA and BPA significantly elevated the expression of IL-5 and IL-13 compared with either OVA alone or BPA alone. These Th2-derived cytokines are key mediators in the symptoms of asthma and are critical for the recruitment and survival of eosinophils [22], the production of antigen specific antibodies [20], and the production of mucous cells, such as goblet cells, in the bronchial epithelium [21]. In this study, a significant correlation was observed between IL-5 and eosinophils (Fig. 5a) and between IL-13 and eosinophils (Fig. 5b) in BALF. Therefore, the occurrence of airway injury during co-treatment may be due to activated eosinophils via Th2-associated effector molecules.

Eosinophils are proinflammatory granulocytes implicated in host immunity to parasitic infections and allergic diseases such as asthma, allergic rhinitis and atopic dermatitis [23]. Appropriate stimuli such as immunoglobulins [24], cytokines [25] and lipid mediators, including leukotrienes [26] and platelet-activating factors [27], induce degranulation of toxic eosinophil granule proteins. Subsequent tissue damage induced by eosinophil granule proteins and superoxides contributes to the airway inflammation associated with such diseases [23, 28].

When we investigated OVA-specific immunoglobulin production, we detected adjuvant effects of BPA on IgG1 production. IgG1 can contribute to antigen-specific eosinophil degradation via FcγRII on the surface of eosinophils [29]. Therefore, increased levels of OVA-specific IgG1 might be related to the heightened levels of cytokines and lung eosinophilia.

Macrophages are key cells in innate immune responses in the lung: they are among the most abundant cells and one of the first to encounter allergens and other threats to homeostasis [30]. Depending on the signals received, macrophages can be pro- or anti-inflammatory, immunogenic or tolerogenic, and can destroy or repair tissue [31]. Our present results show that administration of BPA induced proinflammatory cytokine and chemokine levels. These results provided further confirmation that BPA exacerbates inflammation and airway symptoms in asthmatic mice.

## Conclusions

In conclusion, in this novel study on adult murine asthma model, we showed an aggravating effect of BPA on eosinophil infiltration and airway inflammation by increasing levels of Th2 cytokines and chemokines. In addition, BPA had an enhancing effect on the more activated macrophages, as it increased proteins expression associated with inflammation by these cells. This study confirms that BPA exerts potential effects on allergic inflammation in allergic asthmatics vs. healthy mice and supports findings of previous epidemiologic studies on populations, which indicate BPA can exacerbate allergic airways inflammation. The experimental findings in the present study may serve as a warning about the ill effects of high doses of BPA on the adult human respiratory system.

## Abbreviations

BALF: bronchoalveolar lavage fluid
BPA: bisphenol A
H and E: hematoxylinand eosin
Ig: immunoglobulin
IL: interleukin
CCL2: monocyte chemotactic protein-1
CCL3: macrophage inflammmatory protein-1α
CXCL1: keratinocyte chemoattractant
OVA: ovalbumin
PAS: periodic acid-Schiff
